# Evaluation of the GSP Creatine Kinase-MM Assay and Assessment of CK-MM Stability in Newborn, Patient, and Contrived Dried Blood Spots for Newborn Screening for Duchenne Muscular Dystrophy

**DOI:** 10.3390/ijns8010012

**Published:** 2022-01-28

**Authors:** Brooke A. Migliore, Linran Zhou, Martin Duparc, Veronica R. Robles, Catherine W. Rehder, Holly L. Peay, Katerina S. Kucera

**Affiliations:** 1 RTI International, Research Triangle Park, Durham, NC 27709, USA; bmigliore@rti.org (B.A.M.); lzhou@rti.org (L.Z.); mduparc@rti.org (M.D.); vrobles@rti.org (V.R.R.); hpeay@rti.org (H.L.P.); 2 Department of Pathology, Duke University, Durham, NC 27710, USA; catherine.rehder@duke.edu

**Keywords:** creatine kinase-MM, dried blood spots, Duchenne Muscular Dystrophy, newborn screening, assay performance, sample storage, analyte stability

## Abstract

Duchenne Muscular Dystrophy (DMD) is a fatal X-linked disorder with a birth prevalence of 19.8:100,000 males worldwide. Elevated concentration of the muscle enzyme creatine kinase-MM (CK-MM) allows for presymptomatic screening of newborns using Dried Blood Spots (DBS). We evaluated imprecision and carryover of the FDA-approved PerkinElmer GSP Neonatal CK-MM kit over multiple runs, days, and operators, followed by quantification of CK-MM loss in stored newborn, contrived, and non-newborn patient DBS resulting from exposure to ambient versus low humidity (50-day trial), and high humidity and high temperature (8-day trial). Imprecision %CV was ≤14% for all verification comparisons and over 6 months of testing. On average, the mean CK-MM recovery after 50 days was >80% of initial concentration for all sample types stored in low humidity and <80% in ambient humidity. After 8 days of storage in high humidity and high temperature, the mean recovery for newborn samples was <80%. Verification results for the GSP Neonatal CK-MM assay were concordant with kit parameters and the assay performed consistently over 6 months. CK-MM degradation in ambient storage can be mitigated by reducing exposure to humidity. Assessment of DBS shipping and storage conditions is recommended prior to implementing DMD screening.

## 1. Introduction

Duchenne Muscular Dystrophy (DMD) is an X-linked degenerative neuromuscular disorder causing severe progressive muscle loss and premature death by the mid-30s [1]. It is caused by mutations in the gene coding for the protein dystrophin, an important component within muscle tissue that provides structural stability to cells by participating in linking the cytoskeleton to the extracellular matrix [2,3]. The birth prevalence of DMD is 19.8:100,000 males worldwide [4], while females are typically asymptomatic carriers or have mild muscle and cardiac symptoms [5,6]. Females who manifest DMD are exceedingly rare [7]. 

DMD symptoms usually manifest between the ages of 2 and 3 with mean age of diagnosis at 4 years (± 2.3 years) [8]. A majority of patients become nonambulatory by age 15 years, require ventilatory support by age 20, and up to 60% of patients die by age 30 from progressive cardiac and respiratory complications [9]. Genetic testing in the newborn (NB) period is typically not considered unless family history suggests risk of disease; however, recent and emerging treatments for DMD [10] underscore the need to evaluate the potential importance of presymptomatic identification and access to early treatment. 

The creatine kinase (CK) enzyme leaks from muscle cells into the bloodstream after muscle damage, including damage caused by muscular dystrophies. Several newborn screening (NBS) pilot studies have used quantitative detection of CK in DBS to identify newbrons with DMD [11]; however, total CK-based screening detects the activity of all three isoenzymes (CK-MM, CK-MB, and CK-BB) and is therefore nonspecific. Of the three CK isoforms, CK-MM is found predominantly in the skeletal muscle cells and is typically highly elevated in DMD patients, especially early in life [12,13,14]. Quantitative detection of CK-MM in DBS improves specificity of identification of infants with DMD [15,16]; however, some risk of false-positive results remains, as CK-MM may also be elevated in DBS due to other neuromuscular diseases or birth trauma. Conversely, CK-MM may not be elevated in all DMD patients immediately after birth [11] or may degrade prior to testing, leading to false-negative results.

Sample storage conditions affect the stability of some biomarkers used in NBS [17]; therefore, program-specific effects of specimen transportation and storage conditions on analyte stability should be evaluated prior to testing [18]. Time-dependent CK-MM degradation in DBS is related to sample storage temperature and humidity, necessitating pre-implementation and seasonal assessment of CK-MM degradation by NBS programs, particularly in locations experiencing permanent or seasonal high heat, high humidity, and longer DBS transportation or storage times. We assessed CK-MM stability in NB and non-NB specimens and the effects of common transportation and storage conditions on CK-MM degradation in DBS to inform the implementation of DMD screening by NBS programs. 

## 2. Materials and Methods

### 2.1. Testing Instrument and Assay Kit

Testing was performed on one Genetic Screening Processor (GSP) (#2021-0010) using the FDA-approved GSP Neonatal CK-MM kit (#3311-001U, PerkinElmer), as previously described [19,20]. 

### 2.2. Specimens

Newborn (NB) male and female deidentified residual NBS DBS from the North Carolina State Laboratory of Public Health (NCSLPH) were used in the 50- and 8-day stability experiments (*n* = 500 and *n* = 30, respectively). Only samples within the kit specifications (collected < 72 h after birth, > 1500 g birthweight, > 28 weeks gestational age) were included. NB samples were transported from birthing hospitals to NCSLPH, located in Raleigh, NC, in mailing envelopes or boxes without desiccant or cold packs. Once received, specimens were processed for routine NBS and stored in airtight bins with desiccant at ambient temperature until use. At study commencement, NB specimens were no more than 10 days old from date of collection. Muscular Dystrophy (MD) DBS were created from liquid venous blood (EDTA) of 15 non-newborn deidentified donors, aged 9 to 25 years, with neuromuscular disorders known to be associated with elevated CK-MM from Duke Children’s Neuromuscular Clinic. Lab-Contrived (LC) DBS included prototype specimens from the Centers for Disease Control and Prevention (CDC) (*n* = 5) (manuscript in preparation), and DBS prepared in our lab (*n* =11) using whole blood (Zen-Bio, #SER-WB10ML) with CK-MM (SigmaAldrich #9858 (SigmaAldrich, St. Louis, MO, USA)) serially diluted, then applied to filter paper. Quality Control (QC) materials included kit calibrators and kit controls (QC1 low, QC2 medium, and QC3 high) (#3311-001U, PerkinElmer), blank Whatman 903 filter paper, an LC normal control (QC4), and a CDC prototype DBS, implemented as a positive control in the stability studies (QC5). QC4 DBS were prepared using venous blood (EDTA) from a healthy individual (Zen-Bio, #SER-WB10ML (Zen-Bio, Durham, NC, USA)). DBS cards were prepared in a biosafety hood, as described previously [21]. Non-NB DBS were stored with desiccant at −20 °C until use. Unspotted Whatman 903 filter paper was used as a blank for carryover detection. All samples were tested as 3.2 mm punches. 

### 2.3. Sample Storage Conditions

In the 50-day trial, change in CK-MM concentration was monitored in samples stored in (1) ambient temperature and ambient humidity (MD, LC) and (2) ambient temperature and low humidity (NB, MD, LC). The low-humidity ambient temperature environment was an airtight bin (28.5-quart Superior Storage Box) with desiccant (Desi-can, #420460000). The ambient humidity environment was an open bin without desiccant. In the 8-day high-humidity and high-temperature trial, CK-MM concentration was monitored in NB, MD, and LC DBS stored in an unsealed bag in an unconditioned outdoor enclosure in mid-August in central North Carolina to mimic potential extreme DBS shipping conditions. Temperature and humidity were recorded daily for each condition using a digital thermometer and a hygrometer.

### 2.4. Assay Performance Verification

Kit controls (QC1-QC3) and the LC control (QC4) were used to assess precision and carryover on one GSP instrument. In the assay verification, 23 plates were tested over 5 days as follows: 3 plates containing QC in replicates of 5, 20 plates with QC in duplicate, 2 plates were tested by 2 operators, and carryover was assessed on 2 plates over 2 days with a DBS with high CK-MM concentration preceding the blank. After assay verification, precision and carryover were monitored with QC1-QC4 and a CDC prototype sample with high CK-MM concentration (QC5) in duplicate for 6 months.

### 2.5. CK-MM Stability in DBS Samples

In the 50-day study, after initial testing at timepoint 0, MD and LC samples were stored in low or ambient humidity and tested every 10 days for 50 days. Because of the limited amount of each NB sample, the 500 NB samples used in the trial were tested at timepoint 0, then split into four groups, stored in low-humidity conditions, and tested in a staggered design spanning the duration of the 50-day time course, such that each NB sample was tested at three timepoints and 200 samples were tested per timepoint. Group 1 (*n* = 200) was tested at timepoints 0, 40, and 50; group 2 (*n* = 100) at timepoints 0, 20, and 30; group 3 (*n* = 100) at timepoints 0, 10, and 30; group 4 (*n* = 100) at timepoints 0, 10, and 20. In the 8-day high-temperature and high-humidity trial, 30 NB samples were tested in singlicate and 15 MD and 15 LC samples were tested in duplicate at timepoints 0, 2, 4, 6, and 8 days. CK-MM degradation assessment was performed by comparing CK-MM concentrations at each time point to timepoint 0 to identify conditions at which <80% of the starting CK-MM concentration was recovered, as performed in previous studies [PerkinElmer GSP Neonatal Creatine Kinase-MM kit 3311-001U instructions for use version 1.19].

### 2.6. Statistical Analysis

The mean, standard deviation, and Coefficient of Variation (CV) for the QC materials were calculated using the SpecimenGate QC module (PerkinElmer) and Excel. Statistical analyses for the stability study were conducted with R version 4.0.4. The Shapiro–Wilks test of normality, from the *shapiro.test()* function in the *stats* package, was used to assess the normality of CK-MM concentration distribution among the NB specimens. The Kruskal–Wallis H test, from the *kruskal.test()* function in the *stats* package, was used to compare the initial concentrations of NB sample groups. Generalized estimating equations (GEEs) were used to model the population-averaged mean proportion of initial concentration, with storage condition (categorical variable with 1 = ambient humidity and 0 = low humidity), time (in days), sample type (two categorical dummy variables for LC and MD sample status), and initial concentration as main effects. A separate model was fit for the interaction of time with each of storage condition, sample type, and initial concentration. A Gaussian distribution with a log link function was used, along with an unstructured correlation structure. Statistical significance was evaluated using the robust Z-score. Estimation was performed using the *gee* package.

## 3. Results

### 3.1. Assay Verification and Performance 

Within-run, plate-to-plate, and inter-operator precision comparisons were ≤14% CV and were concordant with the kit parameters (Table 1) [PerkinElmer GSP Neonatal Creatine Kinase-MM kit 3311-001U instructions for use version 1.19]. Additional assay performance data collected for calibrators (data not shown) and QC specimens (Table 1) over 6 months of CK-MM screening [22] as a part of the Early Check study [23] were ≤11.5% CV. Three kit lots were used in the 6-month evaluation. Carryover results were below the 29.2 ng/mL kit lower limit of linearity/measuring range in both the assay verification (*n* = 2) and 6-month evaluation (*n* = 82) (carryover concentration range 0–7 ng/mL). 

### 3.2. 50-Day CK-MM Stability Comparison in Ambient and Low Humidity

The 500 samples used in the 50-day study had been transported from collection sites to the NCSLPH in February, where the mean temperature and humidity in Raleigh were 5.6 °C and 76%, respectively (www.timeanddate.com, accessed on 11 January 2022). Over the 50-day stability trial, the mean difference between the two humidity storage conditions was 27.2% and the storage temperatures remained within 2.3 °C for both humidity conditions. In low-humidity storage, the mean temperature was 18.7 °C (range 17.7–19.4 °C), and the mean humidity was 16.5% (range 12–23%). In ambient humidity storage, the mean temperature was 19.4 °C (range 18.5–20.0 °C) and the mean humidity was 43.7% (range 30–55%).

**Figure 1:**
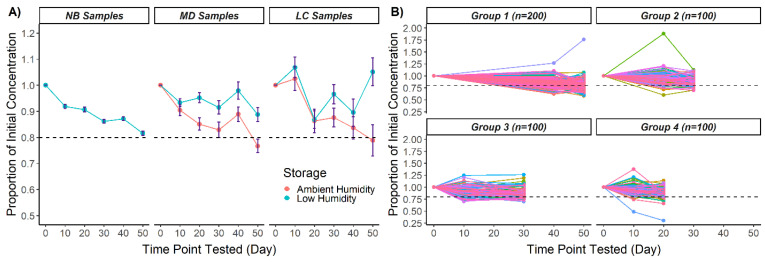
CK-MM recovery over 50 days in ambient and low humidity in three DBS specimen types (NB, MD, LC): (**A**) Mean proportions of initial CK-MM concentrations. Error bars represent ± standard error of the mean. (**B**) Proportion of CK-MM recovery for individual NB DBS. The 500 NB specimens were split into four groups after timepoint 0. CK-MM measurements were taken at three testing timepoints per group, with individual DBS designated by a line. Two hundred DBS from one or more groups were tested in low humidity at each testing timepoint past timepoint 0. Dotted lines in all panels represent 80% of initial concentration.

The 50-day stability trial assessed CK-MM recovery in 500 NB samples, 16 LC samples, and 15 MD samples (Table 2). CK-MM recovery at each timepoint was measured as the proportion of the initial CK-MM concentration at time 0. On average, >80% of the starting CK-MM concentration was recovered after 50 days for all specimen types stored in low humidity (81.6 ± 0.8% NB, 88.8 ± 2.8% MD, and 105.1 ± 5.4% LC). For LC and MD specimens stored in ambient humidity, the mean CK-MM recovery at 50 days was <80% (76.7 ± 2.6% MD and 78.8 ± 6% LC) (Figure 1A, Table S1).

The NB DBS in each group were tested at three timepoints and 200 DBS from one or more groups were tested at each timepoint (Figure 1B). No significant differences were found among the starting concentrations of the four NB groups (Kruskal–Wallis H test, H = 0.805, 3 d.f., *p* = 0.848); thus, the initial CK-MM concentration was balanced among the groups. The 200 NB specimens tested at each timepoint are representative of the whole 500 NB specimen population. 

Even though the aggregated CK-MM concentration was above 80% after 50 days of storage in low humidity, for a proportion of specimens, CK-MM dropped below 80% at some point in the 50-day trial. At 50 days, CK-MM degraded below 80% of the initial concentration in 42% of NB DBS (*n* = 84), indicating that the risk of significant CK-MM degradation over time remains even if humidity is removed with desiccant. The combined proportions of samples with <80% CK-MM recovery after 50 days were 33.2% in low humidity (NB, MD, and LC) and 70.4% in ambient humidity (MD and LC) (Table 3).

GEEs were used to investigate whether CK-MM degradation over time was related to the storage conditions, initial concentration, or sample types (Table 4). The observation that CK-MM recovery over time is decreased in ambient humidity compared to low humidity (Figure 1) was supported (GEE1 in Table 4). The interaction between the initial concentration (for NB, MD, and LC specimens combined) and time (GEE2 in Table 3) suggests that samples with higher initial concentrations had greater CK-MM percent recovery over time compared to samples with lower concentrations. When examining the interaction between sample status and time, the range of CK-MM concentrations in LC and MD DBS was wider relative to the population from which the NB samples were obtained. Therefore, the interaction between MD sample status and time could largely be a function of the generally higher initial CK-MM concentrations and the interaction between LC sample status and time could be obfuscated by the distribution of the CK-MM concentrations across the samples. Finally, CK-MM recovery over time was greater (indicating observed slower degradation) for MD samples compared to NB samples. The interaction between time and LC status was weaker (GEE3 Table 4). Taken together, the interaction between MD sample status and time could result from the generally higher initial CK-MM concentrations of the MD compared to the NB samples. 

### 3.3. 8-Day CK-MM Stability in High Humidity and High Temperature

**Figure 2:**
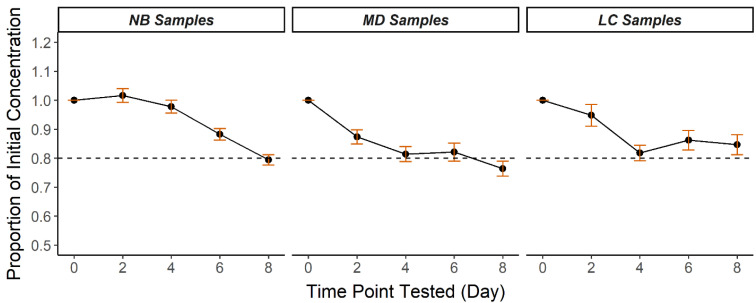
Mean CK-MM recovery over 8 days in high heat and humidity in three DBS specimen types (NB, MD, LC). Results are calculated mean proportions of initial CK-MM concentrations at each timepoint. Error bars represent ± standard error of the mean. Dotted lines represent 80% of initial concentration.

A high-humidity, high-temperature trial was conducted over 8 days in mid-August in central North Carolina using 30 NB, 15 MD, and 15 LC DBS to simulate conditions that samples might be exposed to during transportation (Table 2). The 30 NB samples used in the 8-day study were transported from collection sites to the NCSLPH in August, where the mean temperature and humidity were 25.6 °C and 82% (www.timeanddate.com accessed on 11 January 2022) In the 8-day trial, the mean storage temperature was 29.1 °C (range 23.4–34.9 °C) and the mean humidity was 69.0% (range 55.1–82.8%). In only 8 days at these conditions, CK-MM degradation occurred to an extent not seen until much later in the 50-day trial. After 8 days in high temperature and humidity, the mean percent recovery was 79.4 ± 1.8% NB, 76.4 ± 2.6% MD, and 84.7 ± 3.4% LC (Figure 2, Table S2). At day 8 of the trial, the proportion of DBS with CK-MM concentration <80% of the starting concentration was 73.3% (*n* = 22/30) NB, 66.7% (*n* = 10/15) MD, and 60.0% (*n* = 9/15) LC. 

## 4. Discussion

Several DMD screening pilot studies worldwide have used quantitative detection of CK in DBS to identify DMD cases presymptomatically [11]. More recently, studies have piloted screening with quantitative detection of the CK-MM isoform as a specific biomarker of muscle damage [16,22,24] using the FDA-approved GSP neonatal CK-MM kit (#3311-001U, PerkinElmer). Our assay verification results and assay performance over 6 months indicate that the Neonatal CK-MM kit performed as expected for measuring CK-MM in DBS [19] and that %CVs were similar to other GSP-based NBS assays including thyroid stimulating hormone (TSH), 17-hydroxyprogesterone (17-OHP), immunoreactive trypsin (IRT), total thyroxine (T4) and galactose, galactose-1-phosphate uridyl transferase (GALT) [25]. For assay verification and QC purposes, we created additional sets of DBS cards using blood from non-NB patients and from serial dilutions of blood spiked with purified CK-MM. CK-MM remained stable in the non-NB DBS stored with desiccant in −20 °C over 6 months of testing, indicating that these samples can serve as QC materials for screening, competency assessment, or proficiency testing in cases where NB DBS are not available.

As for many other analytes and as reported previously for CK-MM-based screening for DMD [19,20], understanding factors unrelated to DMD that affect CK-MM concentration in NB DBS is essential to ensure high sensitivity and specificity of NBS. We assessed the recovery of the CK-MM analyte in DBS over time in humidity and temperature conditions typical for NBS programs. While cold storage is ideal for preventing CK-MM degradation for several years [20], removing humidity with desiccant may be used to improve stability when cold storage is impractical, such as during DBS shipping and processing. In this study, loss of CK-MM over the 50-day trial was on average sufficiently reduced by removing humidity; however, for 42% of NB DBS stored with desiccant, CK-MM concentration decreased by ≥20% by day 50, indicating that degradation in stored DBS still contributes some risk for false-negative results, and removing humidity alone is not a sufficient solution for long-term storage. 

This stability study builds on the 34-day study by Moat et al. that evaluated four levels of contrived DBS samples at four temperatures (−20 °C, 4 °C, room temperature, and 37 °C) and three humidity levels (low, ambient, and high) [19]. In our study, in addition to contrived DBS, we included patient (MD) and newborn (NB) specimens and evaluated CK-MM degradation in temperatures and humidity that more closely resemble real-life transportation and storage conditions experienced by NBS programs and research studies [22]. Our finding that reducing humidity in ambient temperature results in recovery of >80% of starting CK-MM concentration over 50 days aligns with the 90% recovery in room temperature and low humidity after 34 days reported by Moat et al. Both studies observed <80% recovery in ambient conditions and faster degradation at high humidity and high temperature [19]. 

Substantial loss of analyte due to heat and humidity occurs for several other analytes already tested by various NBS programs. Summarizing results from 30 ± 5-day stability trials, Adam et al. reported >20% analyte loss for 27 and >90% analyte loss for 7 of the 34 studied markers of inborn errors [17]. The loss of CK-MM observed in our study (>20% analyte loss over 50 days at ambient humidity and temperature and >20% loss over 8 days in high humidity and high temperature) is in line with the rate of degradation of some of the more severely affected analytes from Adam et al. [17]. We note, that the temperature and humidity tested by Adam et al. (37 °C and >90%) were higher, and more similar to Moat et al. [19], than our 8-day trial conditions (25.6 °C and 82%), and would likely cause even greater CK-MM loss.

Limitations: Due to limited specimen availability, NB specimens were not evaluated at storage in ambient temperature and ambient humidity; however, the MD and LC comparisons suggest that the difference in CK-MM degradation in NB specimens stored at the two humidity conditions would be similar to the non-NB samples. Only one GSP instrument was used in the study; thus, multi-instrument variability was not assessed. While the manufacturer indicates that instrument-to-instrument variation is well controlled [PerkinElmer GSP Neonatal Creatine Kinase -MM kit 3311-001U instructions for use version 1] instrument comparison should be included as a part of assay verification by programs that use multiple units. NB specimens were not tested at timepoint 0 due to shipping from birthing hospitals and the need to perform routine state NBS first. The study results suggest that some degradation likely occurred during transport, particularly for specimens collected in the summer. Upon arrival to NCSLPH, all specimens are kept in sealed boxes with desiccant; thus, degradation during short-term storage was likely minimal.

## 5. Conclusions

We show that the performance of the GSP-based CK-MM assay is similar to other NBS tests and, like some other NBS analytes, degradation of CK-MM is a concern for NBS for DMD, particularly in locations with seasonal high heat and humidity. Thus, evaluation of program-specific DBS storage and shipping conditions—namely humidity, temperature, and storage/shipping time—and implementation of appropriate methods to mitigate the risk for false-negative results due to CK-MM degradation prior to implementing NBS for DMD are advised. If CK-MM degradation is substantial, separate cutoffs may be necessary for seasonal effects or delayed testing.

## Figures and Tables

**Table 1:** CK-MM assay precision; mean concentrations are in ng/mL.

Sample	Within-Run (*n* = 55)			Inter-Operator (*n* = 4)			Plate-to-Plate (*n* = 23)			6-Months Evaluation *		
	Mean	SD	CV (%)	Mean	SD	CV (%)	Mean	SD	CV (%)	Mean	SD	CV (%)
**QC1**	111.3	11.1	10.0	107.5	8.7	8.1	113.1	9.3	8.2	116.0	9.7	8.4
**QC2**	385.1	39.5	10.3	374.8	32.7	8.7	389.6	31.1	8.0	387.0	24.0	6.2
**QC3**	1641.2	156.0	9.5	1549.5	39.2	2.5	1661.0	128.2	7.7	1625.0	123.5	7.6
**QC4**	83.1	11.7	14.0	83.0	4.6	5.5	83.8	8.8	10.5	84.0	9.7	11.5
**QC5**	-	-	-	-	-	-	-	-	-	3606.0	383.0	10.6

* Sample sizes for QC1-QC5 for the 6-months evaluation were: QC1 *n* = 103, QC2 *n* = 96, QC3 *n* = 104, QC4 *n* = 164, QC5 *n* = 164.

**Table 2:** Baseline CK-MM concentration (Conc.) for LC, MD, and NB samples.

Sample Type	*n*	Mean Conc. (ng/mL)	SD Conc. (ng/mL)	Min. Conc. (ng/mL)	Max. Conc. (ng/mL)	Median Conc. (ng/mL)	IQR Conc. (ng/mL)
**LC Samples**							
50-Day Study	16	2927.84	3163.32	66	12,697	1827.50	3004.50
8-Day Study	15	3365.20	4622.14	78	19,069	1975.00	3360.50
**MD Samples**							
50-Day Study	15	2465.03	1963.84	300	8368	1931.50	1538.00
8-Day Study	15	2743.33	2200.62	371	9372	2500.00	2362.25
**NB Samples**							
50-Day Study	500	379.54	283.91	10	2820	315.00	293.25
8-Day Study	30	256.93	180.41	70	846	188.50	164.50

**Table 3:** Proportion (Prop.) and number of specimens with <80% CK-MM recovery over 50 days.

Sample Tpe	Day 10		Day 20		Day 30		Day 40		Day 50	
	Count	Prop.	Count	Prop.	Count	Prop.	Count	Prop.	Count	Prop.
**LC Samples**										
Low Humidity	2	0.125	6	0.375	4	0.250	2	0.133	6	0.375
Ambient Humidity	2	0.125	7	0.438	7	0.438	10	0.625	14	0.875
**MD Samples**										
Low Humidity	1	0.067	1	0.067	2	0.133	0	0.000	3	0.200
Ambient Humidity	1	0.067	4	0.267	7	0.467	3	0.200	8	0.533
**NB Samples**										
Low Humidity	22	0.110	29	0.145	37	0.185	52	0.260	84	0.420

**Table 4:** Parameter estimates, standard errors, significance, and confidence intervals for interaction terms estimated using GEE, using a Gaussian distribution with a log-link function. For GEE 3, the *p*-values are compared to a Bonferroni-corrected α = 0.025.

Interaction Type	Estimate	Robust SE	Robust Z	*p*-Value	95% Confidence Interval
**GEE1**					
Ambient Humidity × Time	−2.466 × 10^−3^	8.997 × 10^−4^	−2.740	6.14 × 10^−3^	[−4.23 × 10^−3^, −7.02 × 10^−4^]
**GEE2**					
Initial Concentration × Time	4.034 × 10^−7^	1.648 × 10^−7^	2.448	0.014	[8.05 × 10^−8^, 7.26 × 10^−7^]
**GEE3**					
MD Sample Status × Time	1.829 × 10^−3^	5.728 × 10^−4^	3.193	1.41 × 10^−3^	[7.06 × 10^−4^, 2.95 × 10^−3^]
LC Sample Status × Time	−1.165 × 10^−4^	7.722 × 10^−4^	−0.151	0.880	[−1.63 × 10^−3^, 1.40 × 10^−3^]

## Data Availability

The data are not publicly available due to privacy restrictions.

## Supplementary Materials

Supplementary materials can be found at https://www.mdpi.com/article/10.3390/ijns8010012/s1. Table S1: Proportion of initial CK-MM concentration for LC, MD, and NB samples at time points 10, 20, 30, 40, and 50 in each storage condition. Results are calculated mean proportions of initial CK-MM concentrations with standard error (SE). Table S2: Proportion of initial CK-MM concentration for LC, MD, and NB samples at time points 2, 4, 6, and 8 days in high temperature and high humidity storage. Results are calculated mean proportions of initial CK-MM concentrations with standard error (SE).
