# Supplementary material for: Evaluation of the GSP Creatine Kinase-MM Assay and Assessment of CK-MM Stability in Newborn, Patient, and Contrived Dried Blood Spots for Newborn Screening for Duchenne Muscular Dystrophy
Source: Int J Neonatal Screen. 2022 Jan 28;8(1):12. doi: 10.3390/ijns8010012 (PMC8883886; doi:10.3390/ijns8010012)
Supplement: Supplementary file 1 [file IJNS-08-00012-s001.zip › IJNS-1537717-supplementary.pdf]

Supplementary Table S1: Proportion of initial CK-MM concentration for LC, MD, and NB samples at time points 10, 20, 30, 40, and 50 in each storage condition. Results are calculated mean proportions of initial CK-MM concentrations with standard error (SE).

	LC Samples				*MD Samples*				*NB Samples*	
	**Low Humidity**		**Ambient Humidity**		**Low Humidity**		**Ambient Humidity**		**Low Humidity**	
Time Since Initial (Days)	*Proportion of Initial*	*SE*	*Proportion of Initial*	*SE*	*Proportion of Initial*	*SE*	*Proportion of Initial*	*SE*	*Proportion of Initial*	*SE*
**10**	1.0683	0.0394	1.0247	0.0455	0.9331	0.0149	0.9039	0.0201	0.9184	0.0079
**20**	0.8675	0.0330	0.8630	0.0453	0.9520	0.0189	0.8508	0.0233	0.9065	0.0094
**30**	0.9655	0.0375	0.8757	0.0361	0.9151	0.0255	0.8291	0.0293	0.8614	0.0072
**40**	0.8947	0.0519	0.8365	0.0427	0.9791	0.0330	0.8891	0.0278	0.8714	0.0068
**50**	1.0513	0.0542	0.7884	0.0600	0.8875	0.0275	0.7666	0.0259	0.8159	0.0081

Supplementary Table S2: Proportion of initial CK-MM concentration for LC, MD, and NB samples at time points 2, 4, 6 , and 8 days in high temperature and high humidity storage. Results are calculated mean proportions of initial CK-MM concentrations with standard error (SE).

	LC Samples		*MD Samples*		*NB Samples*	
Time Since Initial (Days)	*Proportion of Initial*	*SE*	*Proportion of Initial*	*SE*	*Proportion of Initial*	*SE*
**2**	0.9483	0.0380	0.8735	0.0246	1.0167	0.0240
**4**	0.8182	0.0269	0.8143	0.0259	0.9782	0.0227
**6**	0.8624	0.0337	0.8213	0.0314	0.8826	0.0199
**8**	0.8467	0.0343	0.7643	0.0257	0.7944	0.0182
